# A Mistake in Dental Legislation

**Published:** 1914-07-15

**Authors:** E. C. Kirk


### A MISTAKE IN DENTAL LEGISLATION.

BY E. C. KIRK, D.D.S., SC.D.

We are informed that the law enacted by the Legislature of Virginia and approved March 14, 1910, governing the practice of dentistry in that state, has been rescinded, or at least that portion of the law embraced in Section 2, which provided that "From and after January 1st, 1914, Anno Domini, the practice of this specialty in this state shall be a branch or specialty of medicine and surgery; and no person, after this act goes into effect shall be given the examination or a certificate required by Section 4 of this act unless he shall first show to the satisfaction of the Examining board provided herein that he has passed the examination provided by law for applicants to practice medicine or surgery, and has received from the Virginia State Board of Medical Examiners the certificate thereof as required by law to be given by them to such applicants." That is to say, the effort to define the nature and status of dentistry by legal enactment and to make dentists out of physicians by the same scheme has met the fate of Belshazzar's kingdom by the analogous method of being weighed in the balances and found wanting.

How long must we suffer delays and obstruction to professional progress by the efforts of purblind enthusiasts who attempt to fit conditions to definitions rather than to adopt the plan of seeking the truth and defining it later in relation to practical results.

The section of the act herein quoted says: "From and after January 1st, 1914, the practice of this specialty (dentistry?) shall be a branch or specialty of medicine and surgery." Beautiful and reassuring as is the truth contained in this legislative pronunciamento, we cannot help asking, What was it before the act, or in what way has it been changed since the passage of the act, or how will it be changed after the reported repeal of the act that gave the dental profession in Virginia a new legal baptism and official designation? What has dentistry ever been, what will dentistry ever be, other than "a branch or specialty of medicine and surgery?" And can any conceivable legislative gyration ever make it anything else than that which it now is and ever has been—a department or specialty of the science and art of healing, *i. e.*, that which we group under the inclusive general term, Medicine?

It seems evident that the purpose of the re-definition of dentistry in Virginia was to enforce the "medical education" of dentists by compelling the prospective applicant for dental license to get his dental education in a medical school. Here, again, is evidence of a fundamental misconception of the broader conception of the term medical. Is not everything taught in a properly conceived and organized dental school, medical in its essence, and its purpose? If not, why not? The dental school in which the therapeutic and prophylactic ideal does not dominate and color all of its teachings would be little more than a technical trade school.

Dental schools, then as a matter of fact, are teaching medicine in so far as it is applicable to the specialty of dentistry, and the line of advance will be to improve and expand the medical ideals of dental teaching. The expectation that the highest efficiency in dental education and practice may be attained through the training afforded by the conventional medical curriculum is a futile one, because in brief terms the medical curriculum does not fit the demands of efficient dental practice. Nor can it be made to do so, for the self-evident reason that the trend of all educational curricula is toward adaptation to special ends, mainly utilitarian, and the medical curriculum will constantly evolve toward the ideal of making better physicians, while that of dentistry will develop toward making more efficient dentists—utilizing for that purpose all of the resources of medical science and art that are adaptable to its purposes.

The rescinding of the dental statute of Virginia is significant of the practical unsoundness of the principle of making dentists through the agency of the medical curriculum.—*Cosmos*

